# Crystal structure and Hirshfeld surface analysis of 2-methyl-3-nitro-*N*-[(*E*)-(5-nitro­thio­phen-2-yl)methyl­idene]aniline

**DOI:** 10.1107/S2056989021000529

**Published:** 2021-01-19

**Authors:** Sevgi Kansiz, Necmi Dege, Seyhan Ozturk, Nesuhi Akdemir, Erdoğan Tarcan, Ali Arslanhan, Eiad Saif

**Affiliations:** a Samsun University, Faculty of Engineering, Department of Fundamental Sciences, Samsun, 55420, Turkey; b Ondokuz Mayıs University, Faculty of Arts and Sciences, Department of Physics, 55139, Samsun, Turkey; c Ondokuz Mayıs University, Faculty of Arts and Sciences, Department of Chemistry, 55139, Samsun, Turkey; d Amasya University, Faculty of Arts and Sciences, Department of Chemistry, Amasya, Turkey; e Kocaeli University, Faculty of Arts and Sciences, Department of Physics, 41100, Kocaeli, Turkey; f Department of Computer and Electronic Engineering Technology, Sana’a Community College, Sana’a, Yemen; g Ondokuz Mayıs University, Faculty of Engineering, Department of Electrical and Electronic Engineering, 55139, Samsun, Turkey

**Keywords:** crystal structure, thio­phene, 5-nitro­thio­phen-2-yl, Schiff base, Hirshfeld surface analysis, hydrogen bonding

## Abstract

The title compound, C_12_H_9_N_3_O_4_S, synthesized by condensation of 5-nitro­thio­phene-2-carbaldehyde and 2-methyl-3-nitro­aniline, crystallizes in the ortho­rhom­bic space group *P*2_1_2_1_2_1_. In the mol­ecule, the aromatic benzene and thio­phene rings are twisted with respect to each other, making a dihedral angle of 23.16 (7)°. In the crystal, mol­ecules are linked by inter­molecular C—H⋯O hydrogen bonds into chains extending along the *c*-axis direction. Weak π–π stacking inter­actions along the *a*-axis direction provide additional stabilization of the crystal structure. The roles of the various inter­molecular inter­actions were clarified by Hirshfeld surface analysis, which reveals that the crystal packing is dominated by O⋯H (39%) and H⋯H (21.3%) contacts. The crystal studied was refined as a two-component inversion twin.

## Abstract 2

The title compound is a Schiff base formed from 5-nitro­thio­phene-2-carbaldehyde and 2-methyl-3-nitro­aniline. In the crystal, the mol­ecules are linked by weak C—H⋯O hydrogen bonds.

## Chemical context   

Bioactivity is an important topic, which includes many areas such as the synthesis of new drugs, creams, agricultural products and so on. In this respect, Schiff bases are organic mol­ecules suitable for bioactivity applications because of the imine bond that increases the lipophilic character of the mol­ecule. The imine bond provides a synthetic route to structural chirality, changes the electronic properties and leads to solubility in different media (Tarafder *et al.*, 2008 ▸). Schiff bases can include heterocycles or amino acid residues and can be easily obtained by the condensation of primary amines with aldehydes or ketones without by-products, thus giving the pure product for biological treatments (Yu *et al.*, 2009 ▸; Lobana *et al.*, 2009 ▸). Many natural products contain thio­phene groups, which lead to pharmacological properties. Thio­phene-containing mol­ecules are used in medicinal chemistry for therapeutic applications (Mishra *et al.*, 2011 ▸). 5-Nitro­thio­phene-2-carbox­aldehyde derivatives exhibit anti­bacterial properties (Foroumadi *et al.*, 2003 ▸). This highly reactive mol­ecule has been used in chemosensor applications (Ye *et al.*, 2019 ▸). In the present study, a new Schiff base, 2-methyl-3-nitro-*N*-[(*E*)-(5-nitro­thio­phen-2-yl)methyl­idene]aniline (I), was obtained in crystalline form from the reaction of 5-nitro­thio­phene-2-carbaldehyde with 2-methyl-3-nitro­aniline.
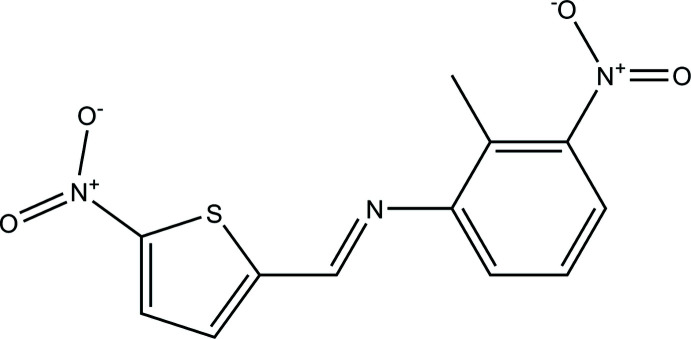


## Structural commentary   

**Figure 1:**
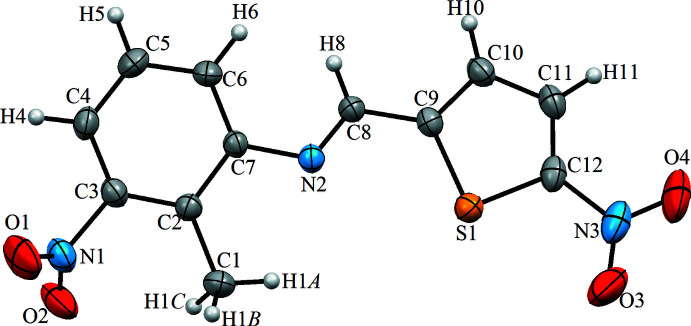
The mol­ecular structure of the title compound with the atom-labelling scheme. Displacement ellipsoids are drawn at the 30% probability level.

The mol­ecular structure of the title compound is shown in Fig. 1 ▸. The mol­ecule adopts the *E* configuration with respect to the C=N bond and the benzene and thio­phene rings form a dihedral angle of 23.16 (7)°. The deviation from planarity can be attributed to packing forces. The nitro group attached to the thio­phene ring is strongly conjugated with the π-system of this ring, as evident from the short N2—C7 distance (see Table 1 ▸). As a result, this nitro group is almost coplanar with the thio­phene ring. The nitro group attached to the benzene ring is twisted by 48.4 (2)° with respect to this ring, and thus the π-conjugation is much weaker in this case. The length of the C8=N2 bond is 1.277 (4) Å, which is consistent with those in the related structures 4-(naphthalen-2-yl)-*N*-[(*Z*)-4-propoxybenzyl­idene]-1,3-thia­zol-2-amine [1.284 (3) Å; Sheakh Mohamad *et al.*, 2020 ▸] and (*E*)-2,4-di-tert-butyl-6-[(3-chloro-4-methyl­phenyl­imino)­meth­yl]phenol [1.278 (4) Å; Kansiz *et al.*, 2018 ▸]. The C9—S1 and C12—S1 bonds in the thio­phene ring are slightly shorter than a standard C*sp* ^2^—S single bond (1.76 Å; Allen *et al.*, 1987 ▸) as a result of the π-conjugation with the double bonds. At the same time, these S—C bonds are longer than those in the structure of 6-[(*E*)-2-(thio­phen-2-yl)ethen­yl]-4,5-di­hydro­pyridazin-3(2*H*)-one [1.691 (3) Å; Daoui *et al.*, 2019 ▸].

## Supra­molecular features   

**Figure 2:**
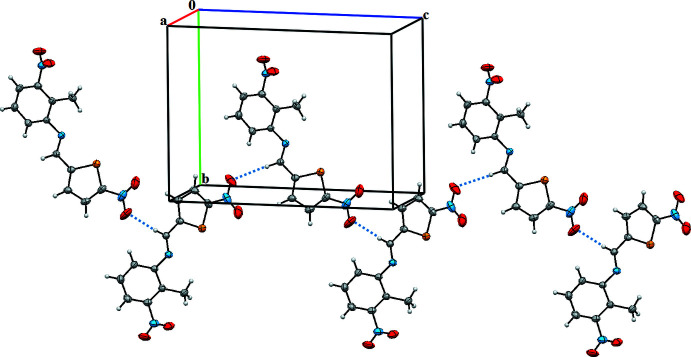
A view of the crystal packing of the title compound parallel to the *bc* plane. C—H⋯O inter­actions are indicated by dotted lines.

In the crystal structure, mol­ecules are connected by weak inter­molecular C8—H8⋯O4^i^ hydrogen bonds into chains stretched along the *c*-axis direction (Table 2 ▸; Fig. 2 ▸). As a result, the mol­ecules form stacks extended along the *a*-axis direction. The shortest inter­centroid separation of 3.603 (2) Å within the stack indicates π–π stacking inter­actions between the benzene and thio­phene rings, which are, however, very weak, since inter­molecular contacts shorter than the sum of van der Waals radii are absent from these stacks.

## Database survey   

A search of the Cambridge Structural Database (CSD, version 5.41, update of November 2019; Groom *et al.*, 2016 ▸) for (*E*)-*N*-[(5-nitro­thio­phen-2-yl)methyl­ene]aniline gave 15 hits including 4-methyl-*N*-[(5-nitro­thio­phen-2-yl)methyl­idene]aniline (EXIWIS; Cai *et al.*, 2011 ▸), *N*-(2-chloro­phen­yl)-1-(5-nitro­thio­phen-2-yl)methanimine (FIBKUZ; Tari *et al.*, 2018 ▸) and 1-(5-nitro-2-thien­yl)-*N*-(2-phen­oxy­phen­yl)methanimine (TONBAB; Tanak *et al.*, 2014 ▸). In FIBKUZ and TONBAB, inter­molecular C—H⋯O hydrogen bonds are important features in the crystal packing, as in the structure of the title compound. In EXIWIS, the C=N bond length [1.277 (2) Å] is the same as in the title compound and longer than in both FIBKUZ [1.265 (6) Å] and in TONBAB [1.261 (4) Å]. The N—O bond lengths in the nitro groups in the title compound are the same within standard deviations as the corresponding bond lengths in all of the reference structures. The C—S bond lengths in EXIWIS, FIBKUZ and TONBAB range from 1.694 (3) to 1.730 (2) Å. The corresponding bond lengths in the title compound fall within these limits.

## Hirshfeld surface analysis   

**Figure 3:**
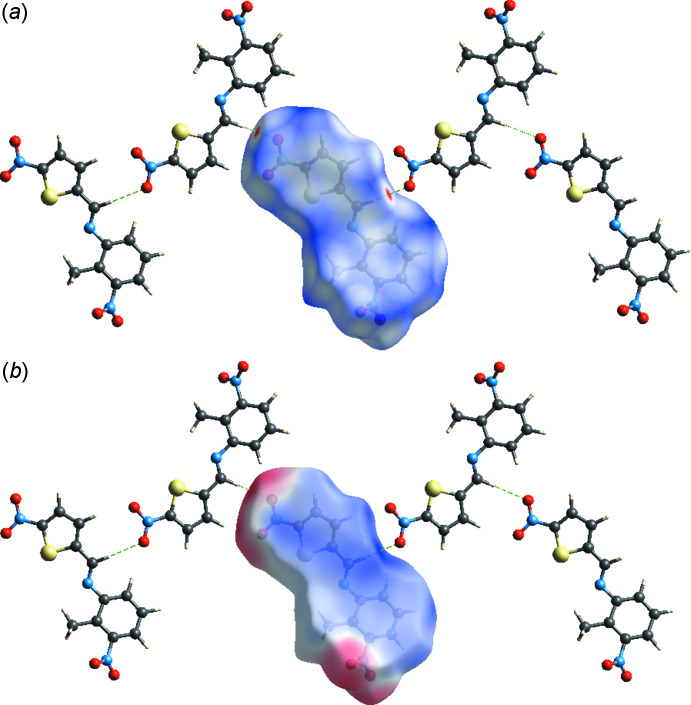
Hirshfeld surfaces of the title compound mapped over (*a*) *d_norm_* and (*b*) electrostatic potential.

**Figure 4:**
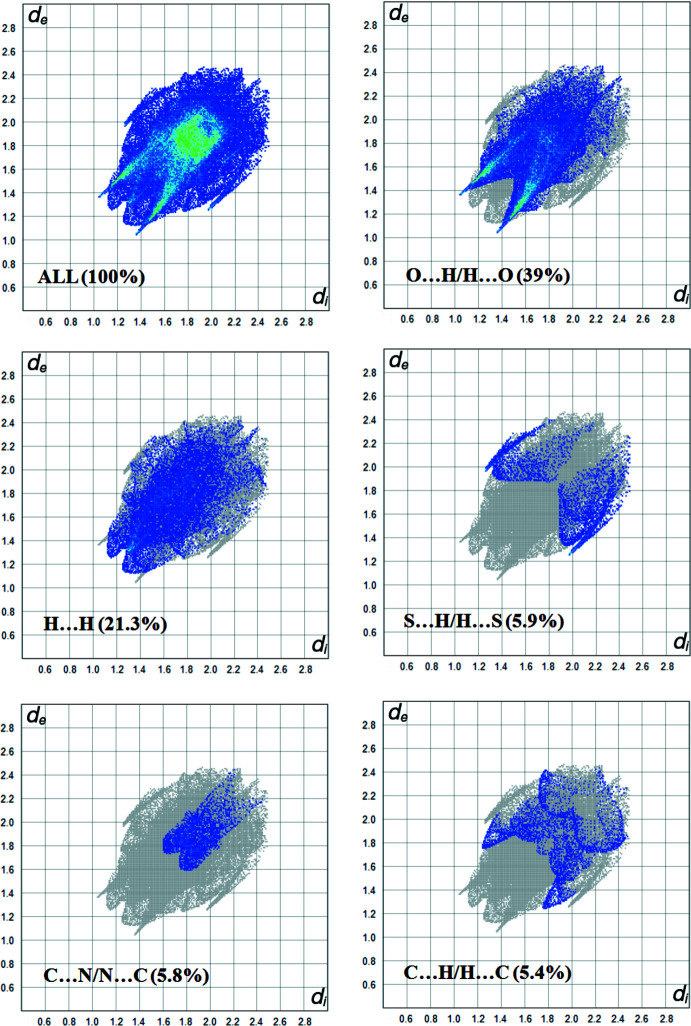
Two-dimensional fingerprint plots for the title compound, with the relative contributions of the atom pairs to the Hirshfeld surface.

The Hirshfeld surface analysis (Spackman & Jayatilaka, 2009 ▸) was carried out using the *CrystalExplorer17.5* (Turner *et al.*, 2017 ▸). The Hirshfeld surface and the associated two-dimensional fingerprint plots were used to qu­antify the various inter­molecular inter­actions in the title compound. The Hirshfeld surfaces mapped over *d*_norm_ and electrostatic potential are illustrated in Fig. 3 ▸. In Fig. 3 ▸ *a*, the red spots correspond to the O⋯H contacts. The electrostatic potential (Fig. 3 ▸ *b*) shows donor (red) and acceptor (blue) regions. The percentage contribution of various inter­actions is shown in the fingerprint plot (Fig. 4 ▸). The most important inter­actions for determining the morphology of the crystal are H⋯H, O⋯H and S⋯H contacts, their individual contributions being 39%, 21.3% and 5.9%, respectively. C⋯N/N⋯C (5.8%) and C⋯H/H⋯C (5.4%) contacts are also observed. The Hirshfeld surface analysis confirms the importance of H-atom contacts in establishing the crystal packing.

## Synthesis and crystallization   

The title compound was prepared by refluxing a solution containing 5-nitro­thio­phene-2-carbaldehyde (0,07 mmol) and 2-methyl-3-nitro­aniline (0,07 mmol) in ethanol (40 ml) for 5 h under stirring. The obtained yellow crystalline material was washed with ethanol and dried at room temperature (yield: 78%, m.p. 433 K). Crystals were grown from a solution in ethanol.

## Refinement   

Crystal data, data collection and structure refinement details are summarized in Table 3 ▸. The C-bound H atoms were placed in idealized positions and refined using a riding model with C—H = 0.93–0.96 Å and *U*_iso_(H) = 1.5*U*_eq_(C-meth­yl) and 1.2*U*_eq_(C) for other C-bound H atoms. The structure was refined as a two-component inversion twin.

## Supplementary Material

Crystal structure: contains datablock(s) I. DOI: 10.1107/S2056989021000529/yk2142sup1.cif

Structure factors: contains datablock(s) I. DOI: 10.1107/S2056989021000529/yk2142Isup2.hkl

Supporting information file. DOI: 10.1107/S2056989021000529/yk2142Isup3.cml

CCDC reference: 2055920

Additional supporting information:  crystallographic information; 3D view; checkCIF report

## Figures and Tables

**Table 1:** Selected bond lengths (Å)

S1—C12	1.714 (4)	N1—O2	1.218 (5)
S1—C9	1.718 (4)	N1—C3	1.474 (5)
N2—C8	1.277 (4)	N3—O3	1.216 (6)
N2—C7	1.411 (4)	N3—O4	1.230 (6)
N1—O1	1.211 (5)	N3—C12	1.423 (5)

**Table 2:** Hydrogen-bond geometry (Å, °)

*D*—H⋯*A*	*D*—H	H⋯*A*	*D*⋯*A*	*D*—H⋯*A*
C8—H8⋯O4^i^	0.93 (4)	2.56 (4)	3.492 (5)	176 (3)

Symmetry code: (i) 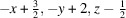.

**Table 3:** Experimental details

Crystal data	
Chemical formula	C_12_H_9_N_3_O_4_S
*M*_r_	291.28
Crystal system, space group	Orthorhombic, *P*2_1_2_1_2_1_
Temperature (K)	293
*a*, *b*, *c* (Å)	7.1335 (4), 11.7297 (6), 15.4593 (7)
*V* (Å^3^)	1293.54 (11)
*Z*	4
Radiation type	Mo *K*α
μ (mm^−1^)	0.27
Crystal size (mm)	0.75 × 0.39 × 0.14

Data collection	
Diffractometer	Stoe IPDS 2
Absorption correction	Integration (*X-RED32*; Stoe & Cie, 2002 ▸)
*T*_min_, *T*_max_	0.839, 0.966
No. of measured, independent and observed [*I* > 2σ(*I*)] reflections	6707, 3930, 2258
*R*_int_	0.049
(sin θ/λ)_max_ (Å^−1^)	0.714

Refinement	
*R*[*F* ^2^ > 2σ(*F* ^2^)], *wR*(*F* ^2^), *S*	0.053, 0.129, 0.91
No. of reflections	3930
No. of parameters	186
H-atom treatment	H atoms treated by a mixture of independent and constrained refinement
Δρ_max_, Δρ_min_ (e Å^−3^)	0.33, −0.19
Absolute structure	Refined as an inversion twin.
Absolute structure parameter	0.59 (15)

Computer programs: *X-AREA* and *X-RED32* (Stoe & Cie, 2002 ▸), *SHELXT2017/1* (Sheldrick, 2015*a* ▸), *SHELXL2017/1* (Sheldrick, 2015*b* ▸), *PLATON* (Spek, 2020 ▸) and *WinGX* (Farrugia, 2012 ▸).

## supplementary crystallographic information

### Crystal data

**Table:**

C_12_H_9_N_3_O_4_S	*D*_x_ = 1.496 Mg m^−^^3^
*M**_r_* = 291.28	Mo *K*α radiation, λ = 0.71073 Å
Orthorhombic, *P*2_1_2_1_2_1_	Cell parameters from 6261 reflections
*a* = 7.1335 (4) Å	θ = 2.2–30.9°
*b* = 11.7297 (6) Å	µ = 0.27 mm^−^^1^
*c* = 15.4593 (7) Å	*T* = 293 K
*V* = 1293.54 (11) Å^3^	Stick, yellow
*Z* = 4	0.75 × 0.39 × 0.14 mm
*F*(000) = 600	

### Data collection

**Table:**

Stoe IPDS 2 diffractometer	3930 independent reflections
Radiation source: sealed X-ray tube, 12 x 0.4 mm long-fine focus	2258 reflections with *I* > 2σ(*I*)
Detector resolution: 6.67 pixels mm^-1^	*R*_int_ = 0.049
rotation method scans	θ_max_ = 30.5°, θ_min_ = 2.2°
Absorption correction: integration (X-RED32; Stoe & Cie, 2002)	*h* = −8→10
*T*_min_ = 0.839, *T*_max_ = 0.966	*k* = −16→12
6707 measured reflections	*l* = −22→20

### Refinement

**Table:**

Refinement on *F*^2^	Hydrogen site location: mixed
Least-squares matrix: full	H atoms treated by a mixture of independent and constrained refinement
*R*[*F*^2^ > 2σ(*F*^2^)] = 0.053	*w* = 1/[σ^2^(*F*_o_^2^) + (0.063*P*)^2^] where *P* = (*F*_o_^2^ + 2*F*_c_^2^)/3
*wR*(*F*^2^) = 0.129	(Δ/σ)_max_ < 0.001
*S* = 0.91	Δρ_max_ = 0.33 e Å^−^^3^
3930 reflections	Δρ_min_ = −0.19 e Å^−^^3^
186 parameters	Absolute structure: Refined as an inversion twin.
0 restraints	Absolute structure parameter: 0.59 (15)

### Special details

**Table:**

Geometry. All esds (except the esd in the dihedral angle between two l.s. planes) are estimated using the full covariance matrix. The cell esds are taken into account individually in the estimation of esds in distances, angles and torsion angles; correlations between esds in cell parameters are only used when they are defined by crystal symmetry. An approximate (isotropic) treatment of cell esds is used for estimating esds involving l.s. planes.
Refinement. Refined as a two-component inversion twin

### Fractional atomic coordinates and isotropic or equivalent isotropic displacement parameters (Å^2^)

**Table:**

	*x*	*y*	*z*	*U*_iso_*/*U*_eq_	
S1	0.63801 (15)	0.83820 (8)	0.62698 (6)	0.0504 (3)	
N2	0.6540 (5)	0.6808 (2)	0.47460 (18)	0.0437 (7)	
N1	0.7061 (6)	0.2794 (3)	0.3994 (3)	0.0637 (10)	
O2	0.8371 (6)	0.2537 (3)	0.4460 (2)	0.0886 (11)	
O1	0.5937 (6)	0.2119 (3)	0.3708 (3)	0.0988 (13)	
C8	0.7015 (6)	0.7831 (3)	0.4567 (2)	0.0432 (8)	
C7	0.6589 (5)	0.5963 (3)	0.4097 (2)	0.0397 (7)	
C9	0.6899 (5)	0.8708 (3)	0.5213 (2)	0.0431 (8)	
C6	0.6287 (6)	0.6186 (3)	0.3229 (2)	0.0487 (9)	
H6	0.610040	0.693392	0.304891	0.058*	
C3	0.6828 (5)	0.4000 (3)	0.3749 (3)	0.0474 (8)	
N3	0.6315 (7)	1.0145 (4)	0.7400 (3)	0.0756 (12)	
C2	0.6860 (5)	0.4834 (3)	0.4389 (2)	0.0423 (8)	
C4	0.6524 (7)	0.4210 (3)	0.2888 (2)	0.0549 (9)	
H4	0.649811	0.361601	0.249035	0.066*	
O3	0.5983 (7)	0.9420 (5)	0.7941 (2)	0.1054 (15)	
C12	0.6570 (6)	0.9799 (3)	0.6526 (2)	0.0537 (10)	
C11	0.7002 (6)	1.0482 (3)	0.5853 (3)	0.0575 (11)	
H11	0.714508	1.126873	0.589073	0.069*	
O4	0.6437 (7)	1.1168 (4)	0.7564 (3)	0.1149 (15)	
C10	0.7204 (6)	0.9856 (3)	0.5095 (3)	0.0520 (10)	
H10	0.751275	1.017854	0.456431	0.062*	
C1	0.7131 (7)	0.4585 (4)	0.5333 (3)	0.0559 (10)	
H1A	0.710488	0.528510	0.565450	0.084*	
H1B	0.614362	0.409413	0.553194	0.084*	
H1C	0.831809	0.421538	0.541751	0.084*	
C5	0.6258 (7)	0.5321 (4)	0.2626 (2)	0.0573 (10)	
H5	0.606041	0.548561	0.204434	0.069*	
H8	0.747 (5)	0.807 (3)	0.403 (2)	0.034 (9)*	

### Atomic displacement parameters (Å^2^)

**Table:**

	*U*^11^	*U*^22^	*U*^33^	*U*^12^	*U*^13^	*U*^23^
S1	0.0561 (5)	0.0487 (5)	0.0465 (4)	−0.0019 (5)	−0.0005 (5)	−0.0003 (4)
N2	0.0474 (16)	0.0390 (15)	0.0448 (15)	−0.0002 (14)	0.0051 (14)	−0.0033 (12)
N1	0.075 (3)	0.0406 (18)	0.075 (3)	0.0010 (19)	0.002 (2)	−0.0039 (17)
O2	0.110 (3)	0.0465 (17)	0.109 (3)	0.012 (2)	−0.021 (3)	0.0103 (18)
O1	0.120 (3)	0.0470 (17)	0.130 (3)	−0.0226 (19)	−0.015 (3)	−0.009 (2)
C8	0.052 (2)	0.0367 (18)	0.0407 (18)	0.0013 (15)	0.0051 (16)	0.0002 (14)
C7	0.0433 (19)	0.0334 (15)	0.0424 (17)	−0.0017 (16)	0.0050 (16)	−0.0029 (13)
C9	0.042 (2)	0.0383 (17)	0.0490 (19)	0.0033 (14)	−0.0003 (15)	−0.0014 (15)
C6	0.062 (2)	0.0445 (18)	0.0400 (18)	0.007 (2)	−0.0010 (19)	0.0059 (14)
C3	0.050 (2)	0.0368 (17)	0.055 (2)	0.0010 (15)	0.0040 (19)	0.0005 (17)
N3	0.070 (2)	0.093 (3)	0.063 (2)	0.006 (3)	−0.006 (2)	−0.032 (2)
C2	0.045 (2)	0.0399 (18)	0.0423 (18)	−0.0028 (15)	0.0037 (15)	−0.0006 (15)
C4	0.064 (3)	0.050 (2)	0.050 (2)	0.002 (2)	0.005 (2)	−0.0148 (17)
O3	0.131 (4)	0.134 (4)	0.052 (2)	0.012 (3)	0.006 (2)	−0.010 (2)
C12	0.050 (2)	0.056 (2)	0.055 (2)	0.004 (2)	−0.0073 (19)	−0.0194 (18)
C11	0.064 (3)	0.038 (2)	0.070 (3)	0.0038 (18)	−0.004 (2)	−0.0101 (19)
O4	0.141 (4)	0.101 (3)	0.102 (3)	0.004 (3)	−0.007 (3)	−0.063 (3)
C10	0.061 (2)	0.0366 (19)	0.058 (2)	−0.0001 (17)	−0.0024 (19)	0.0013 (17)
C1	0.073 (3)	0.046 (2)	0.048 (2)	0.000 (2)	0.002 (2)	0.0085 (17)
C5	0.073 (3)	0.063 (2)	0.0361 (18)	0.002 (3)	−0.001 (2)	−0.0028 (17)

### Geometric parameters (Å, º)

**Table:**

S1—C12	1.714 (4)	C3—C2	1.392 (5)
S1—C9	1.718 (4)	N3—O3	1.216 (6)
N2—C8	1.277 (4)	N3—O4	1.230 (6)
N2—C7	1.411 (4)	N3—C12	1.423 (5)
N1—O1	1.211 (5)	C2—C1	1.501 (5)
N1—O2	1.218 (5)	C4—C5	1.377 (6)
N1—C3	1.474 (5)	C4—H4	0.9300
C8—C9	1.435 (5)	C12—C11	1.349 (6)
C8—H8	0.93 (4)	C11—C10	1.391 (6)
C7—C6	1.384 (5)	C11—H11	0.9300
C7—C2	1.412 (5)	C10—H10	0.9300
C9—C10	1.376 (5)	C1—H1A	0.9600
C6—C5	1.378 (5)	C1—H1B	0.9600
C6—H6	0.9300	C1—H1C	0.9600
C3—C4	1.371 (5)	C5—H5	0.9300
			
C12—S1—C9	89.25 (18)	C3—C2—C1	123.8 (3)
C8—N2—C7	120.0 (3)	C7—C2—C1	120.8 (3)
O1—N1—O2	124.3 (4)	C3—C4—C5	118.6 (3)
O1—N1—C3	117.3 (4)	C3—C4—H4	120.7
O2—N1—C3	118.4 (4)	C5—C4—H4	120.7
N2—C8—C9	120.5 (3)	C11—C12—N3	126.4 (4)
N2—C8—H8	124 (2)	C11—C12—S1	114.6 (3)
C9—C8—H8	115 (2)	N3—C12—S1	119.0 (3)
C6—C7—N2	123.5 (3)	C12—C11—C10	111.1 (4)
C6—C7—C2	120.6 (3)	C12—C11—H11	124.5
N2—C7—C2	115.8 (3)	C10—C11—H11	124.5
C10—C9—C8	126.9 (4)	C9—C10—C11	112.9 (4)
C10—C9—S1	112.2 (3)	C9—C10—H10	123.5
C8—C9—S1	120.9 (3)	C11—C10—H10	123.6
C5—C6—C7	121.3 (3)	C2—C1—H1A	109.5
C5—C6—H6	119.4	C2—C1—H1B	109.5
C7—C6—H6	119.4	H1A—C1—H1B	109.5
C4—C3—C2	124.5 (3)	C2—C1—H1C	109.5
C4—C3—N1	116.1 (3)	H1A—C1—H1C	109.5
C2—C3—N1	119.4 (4)	H1B—C1—H1C	109.5
O3—N3—O4	123.7 (5)	C4—C5—C6	119.7 (4)
O3—N3—C12	118.6 (4)	C4—C5—H5	120.2
O4—N3—C12	117.7 (5)	C6—C5—H5	120.2
C3—C2—C7	115.4 (3)		
			
C7—N2—C8—C9	177.5 (3)	N2—C7—C2—C3	177.4 (3)
C8—N2—C7—C6	−30.4 (6)	C6—C7—C2—C1	−178.2 (4)
C8—N2—C7—C2	153.2 (4)	N2—C7—C2—C1	−1.7 (6)
N2—C8—C9—C10	−173.9 (4)	C2—C3—C4—C5	1.0 (7)
N2—C8—C9—S1	7.5 (5)	N1—C3—C4—C5	178.7 (4)
C12—S1—C9—C10	0.4 (3)	O3—N3—C12—C11	−177.8 (5)
C12—S1—C9—C8	179.2 (3)	O4—N3—C12—C11	2.5 (8)
N2—C7—C6—C5	−176.7 (4)	O3—N3—C12—S1	0.6 (7)
C2—C7—C6—C5	−0.5 (7)	O4—N3—C12—S1	−179.2 (4)
O1—N1—C3—C4	−47.0 (6)	C9—S1—C12—C11	0.0 (4)
O2—N1—C3—C4	132.2 (4)	C9—S1—C12—N3	−178.6 (4)
O1—N1—C3—C2	130.8 (4)	N3—C12—C11—C10	178.1 (4)
O2—N1—C3—C2	−50.0 (6)	S1—C12—C11—C10	−0.3 (5)
C4—C3—C2—C7	−1.2 (6)	C8—C9—C10—C11	−179.3 (4)
N1—C3—C2—C7	−178.8 (3)	S1—C9—C10—C11	−0.7 (4)
C4—C3—C2—C1	177.9 (4)	C12—C11—C10—C9	0.6 (5)
N1—C3—C2—C1	0.3 (6)	C3—C4—C5—C6	−0.5 (7)
C6—C7—C2—C3	0.9 (6)	C7—C6—C5—C4	0.3 (7)

### Hydrogen-bond geometry (Å, º)

**Table:**

*D*—H···*A*	*D*—H	H···*A*	*D*···*A*	*D*—H···*A*
C8—H8···O4^i^	0.93 (4)	2.56 (4)	3.492 (5)	176 (3)

Symmetry code: (i) −*x*+3/2, −*y*+2, *z*−1/2.
